# Microporous polysaccharide hemosphere absorbable hemostat use in cardiothoracic surgical procedures

**DOI:** 10.1186/s13019-014-0134-4

**Published:** 2014-08-02

**Authors:** Brian A Bruckner, Lance N Blau, Limael Rodriguez, Erik E Suarez, Uy Q Ngo, Michael J Reardon, Matthias Loebe

**Affiliations:** 1 Houston Methodist Hospital, Methodist DeBakey Heart & Vascular Center, Houston, TX, USA

**Keywords:** Bleeding control, Biomaterials, Coagulants

## Abstract

**Background:**

Topical hemostatic agents are used to reduce bleeding and transfusion need during cardiothoracic surgery. We report our experience with Arista® AH Absorbable Hemostatic Particles (Arista® AH), a novel plant-based microporous polysaccharide hemostatic powder.

**Methods:**

Data were retrospectively collected for patients (n = 240) that received cardiothoracic surgery at our institution from January 2009 to January 2013 with (n = 103) or without (n = 137) the use of Arista® AH. Endpoints included protamine to skin closure time (hemostasis time), cardiopulmonary bypass time, quantity of Arista® AH applied, intraoperative blood product usage, intraoperative blood loss, chest tube output 48 hours postoperatively, blood products required 48 hours postoperatively, length of stay in the intensive care unit, 30-day morbidity, and 30-day mortality.

**Results:**

240 patients (176 M: 64 F) underwent 240 cardiothoracic procedures including heart transplantation (n = 53), cardiac assist devices (n = 113), coronary artery bypass grafts (n = 20), valve procedures (n = 19), lung transplantation (n = 17), aortic dissection (n = 8), and other (n = 10). Application of Arista® AH led to significant reduction in hemostasis time versus the untreated control group (Arista® AH: 93.4 ± 41 min. vs. Control: 107.6 ± 56 min., p = 0.02). Postoperative chest tube output in the first 48 hours was also significantly reduced (Arista® AH: 1594 ± 949 mL vs. Control: 2112 ± 1437 mL, p < 0.001), as well as transfusion of packed red blood cells (Arista® AH: 2.4 ± 2.5 units vs. Control: 4.0 ± 5.1 units, p < 0.001). There was no significant difference in 30-day mortality or postoperative complications.

**Conclusion:**

Use of Arista® AH in complex cardiothoracic surgery resulted in a significant reduction in hemostasis time, postoperative chest tube output, and need for postoperative blood transfusion.

## Background

Postoperative bleeding complications are associated with poorer outcomes in cardiac surgery and contribute to excessive overall healthcare costs. The need for intraoperative and postoperative blood products are also associated with potential risks including adverse reactions, transfusion related injury, or infectious transmissions that are significant factors for morbidity or mortality [1]. In their study examining open heart procedures requiring re-exploration for bleeding, Hall et al. described that 66% of cases were from surgical bleeding versus 34% attributed to coagulopathy where no surgical bleeding site was found [2].

Bleeding arises as a result of several aspects inherent to these cardiac procedures, including placement of cardiac suture lines in great vessels or chambers of the heart, as well as creation of high pressure anastomoses. Even greater challenges exist during re-operative procedures as patients present to the surgeon on aspirin, other platelet-inhibiting drugs, heparin, and oral anti-coagulants including Coumadin. Heparinization for cardiopulmonary bypass, coagulopathy from extracorporeal membrane oxygenation, and hypothermia may also present an even greater challenge to hemostasis [3]. Postoperatively, excessive chest tube output from inadequate hemostasis may necessitate higher transfusion rates of blood products [4].

Used as an adjuvant measure in the operating room, topical hemostatic agents have been developed to aid in blood loss reduction within the surgical field, which may also reduce overall bleeding in the postoperative period. It is important to delineate differences in the commercially available surgical sealants and broad field hemostatic agents. Sealants are typically employed to anastomotic suture lines prior to removal of clamps as a preventative measure. Topical hemostatic agents are usually applied to the bleeding area and pressure is applied to promote clotting and stop active bleeding. An ideal agent would be one that has the ability to control both types of bleeding, while not posing adverse effects attributed to its application. Arista® AH (C. R. Bard, Inc. – Davol, Warwick, RI) is a microporous polysaccharide hemosphere (MPH) powder derived from a biologically inert plant source that can be administered to the entire surgical field [5]. It absorbs water and low molecular weight compounds from the blood to concentrate platelets and clotting proteins at its beaded surface while enhancing endogenous clotting processes. It has not only been used in cardiothoracic surgery, but also in general, urology and otolaryngology surgical procedures.

The aim of this retrospective study was to evaluate all of the cardiothoracic surgical procedures conducted at a single center in which Arista® AH was used, as compared to an untreated control population, and to analyze the following endpoints: protamine to skin closure time (hemostasis time), cardiopulmonary bypass time, the quantity of Arista® AH applied, use of intraoperative blood products, intraoperative blood loss, chest tube output at 48 hours postoperatively, blood products required at 48 hours postoperatively, length of stay in the intensive care unit, 30-day morbidity, and 30-day mortality.

## Methods

### Patient population

**Figure 1:**
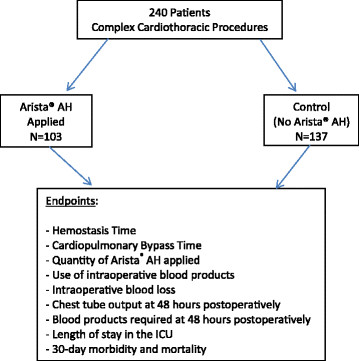
Study design.

This study was approved by the institutional review board at the Houston Methodist Hospital, and patient confidentiality was insured. This retrospective data collection study was designed to gather information from all patients that underwent complex cardiothoracic surgical interventions (cases requiring the use of cardiopulmonary bypass) from January 2009 to January 2013 at the Houston Methodist Hospital (HMH). Sample size for this retrospective analysis was 240 patients. Specifically, we compared 103 patients that received Arista® AH (January 2011 to January 2013) to 137 patients that did not receive Arista® AH (January 2009 to January 2011) (Figure 1). Subjects were identified in one of two ways: 1. Directly from our practice or inpatient data, or 2. Review of medical records within the HMH-Medical Center inpatient list (MethOD) or archived medical records. All surgeries over the study period were performed by two experienced cardiothoracic surgeons with 5+ years of experience post-fellowship starting in 2009. Additionally, during the study period, there were no major changes in technique or protocol in the way operations were conducted, with the exception of topical hemostatic agent choice which occurred in 2011. Complex cardiothoracic surgical procedures included: left, right, or biventricular assist device implantation or explantation, heart transplantation, double lung transplantation requiring cardiopulmonary bypass, aortic reconstructions, coronary artery bypass, and aortic or mitral valve replacements. All patients in the treated and control group received a sealant on aortic or ventricular suture lines, which is standard in our surgical practice. Additionally, it has been our surgical practice following cardiopulmonary bypass cases (all cases included in this study) to apply a topical hemostatic agent as well. Starting in 2011 (the treated group), we only used Arista® AH as a topical hemostatic agent. Furthermore, no significant practice changes were made during the study period as it relates to use of preoperative anticoagulants/procoagulants and perioperative transfusion triggers. The historical control patients from 2009 to 2011 received at least one hemostatic agent FloSeal® (Baxter International, Inc., Deerfield, IL), Gelfoam® with thrombin (Baxter International, Inc., Deerfield, IL), or Surgicel® (Ethicon, Inc., Bridgewater, NJ) without Arista® AH.

### Data collection

All patient records were stratified by age, sex, and complex cardiothoracic surgical procedure. Endpoints evaluated included: protamine to skin closure time (hemostasis time), cardiopulmonary bypass time, the quantity of Arista® AH applied, use of intraoperative blood products, intraoperative blood loss, chest tube output at 48 hours postoperatively, blood products required at 48 hours postoperatively, length of stay in the intensive care unit, 30-day morbidity and 30-day mortality. Morbidity included ventilator dependence (vent requirement >7 days), shock (requiring 2 or more pressors with hypotension), renal failure (new onset requiring dialysis), sepsis (positive blood cultures and hypotension), and stroke (verified by CT scan or MRI scan).

### Scanning electron microscopy (SEM)

To gain visual evidence of the Arista® AH hemostatic mechanism of action, high vacuum SEM was performed on mediastinal clot samples taken from two patients that received Arista® AH. These specimens were obtained from patients intraoperatively, ten minutes after the administration of Arista® AH, and then processed histologically. Specimens were fixed in glutaraldehyde for further analysis. The specimens were subsequently fixed in osmium and coated in gold before undergoing evaluation by SEM, which included acquiring photomicrographs at low and high magnification (up to 1900×) for visual microscopic assessment.

### Surgical technique

**Figure 2:**
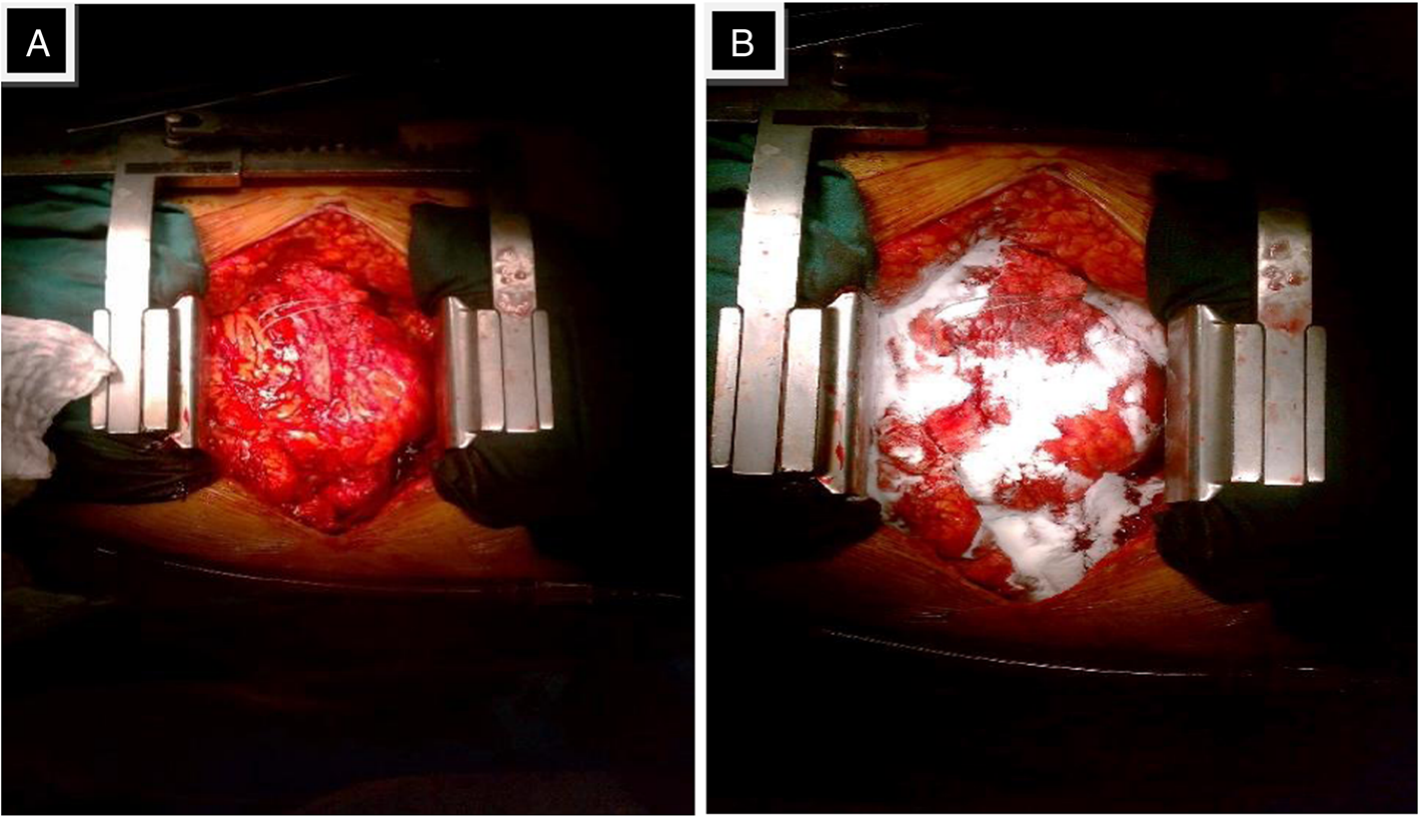
**Intraoperative photographs from a patient undergoing a redo aortic valve replacement procedure.A)** the surgical procedure is complete, including administration of protamine and removal of cannulae. **B)** Arista® AH is applied within the surgical field to areas of active bleeding as a broad field hemostat.

All operations were performed through full median sternotomy except for lung transplantations, which were performed through a clamshell incision. Heparinization for cardiopulmonary bypass was instituted and was reversed by protamine sulfate after weaning from extracorporeal circulation. Among the Arista® AH group patients, Arista® AH (5 gram size) was immediately applied following protamine administration to the entire operative field to address all instances of active bleeding (pulsatile flow, continuous flow, or oozing after application of conventional means including direct pressure, electrocautery, and suture ligation). The number of Arista® AH (5 gram size) applications was dependent upon bleeding and at the discretion of the practicing surgeon. In Figure 2, the intraoperative technique of administration of Arista® AH is demonstrated in a patient that underwent redo aortic valve replacement. Our Arista® AH application technique is to apply the device to the entire surgical field as a broad field hemostatic agent.

### Statistical methods

Data were retrospectively entered in a computerized database and analyzed through SPSS software version 11.0.1 for Windows (SPSS Inc, Chicago, IL) under the guidance of a departmental statistician. Continuous data is presented as the mean +/− standard deviation and compared through the Student *t* test assuming unequal variances. One-way analysis of variance (ANOVA) was employed to disclose significant differences among baseline variables. Categorical data are given as percentages and compared through the Chi Squared test. Tests were two-tailed and Yates correction was applied. Inter-group comparisons were performed in the Arista® AH group by comparing the amount of product used to the data end points using a two sample Wilcoxon Rank Sum Test.

## Results

### Patient population

There was no significant difference in preoperative characteristics (age, sex, procedure type) between the two groups (Table 1). Average patient age in the Arista® AH group was 55 ± 12.6 years (range 15–80) vs. 56 ± 13.7 years (range 20–83) in the control group. Both groups had significantly more men than women, and procedure breakdown was similar with cardiac assist devices and heart transplantations as the most common procedure in both groups.

**Table 1:** Baseline characteristics of the study and control population

**Characteristic**	**Arista group (n = 103)**	**Control group (n = 137)**	***P*** **value**
**Age (yrs.)**	55 ± 12.6 (15–80)	56 ± 13.7 (20–83)	NS
**Sex (M/F)**	79/24	97/40	NS
**Heart transplant**	27	26	NS
**Cardiac assist devices**	52	61	NS
**CABG**	5	15	NS
**Valve procedure**	5	14	NS
**Lung transplant**	8	9	NS
**Aortic dissection**	2	6	NS
Other*	4	6	NS

*Mediastinal explorations, LV aneurysm repairs.

### Endpoints

Intraoperatively (Table 2), there was a significant difference in protamine to skin closure time (hemostasis time) in the Arista® AH group vs. the control group (*p* = .02). There was no significant difference in cardiopulmonary bypass time between the Arista® AH and control group or intraoperative blood product usage. Estimated blood loss was less in the Arista® AH group compared to the control group, but not statistically significant (*p* = .08). Chest tube output 48 hours postoperatively was significantly less in the Arista® AH group compared to the control group (*p <* 0.001) as well as PRBC transfusion requirement (*p <* 0.001) (Table 3). PRBC transfusion requirement was further stratified into need for 1–2 units versus 3 or more units. The control group had a higher percentage of patients (41% vs 34%) requiring 1–2 units and both groups had similar numbers of patients requiring 3 or more units (33% vs 36%) in the first 48 hours following surgery. The Arista® AH group also required fewer platelets and FFP, but these values were not statistically significant. There was no significant difference in ICU stay, mortality (Table 3) or morbidity (defined as ventilator dependence, shock, renal failure, sepsis, or stroke) between the groups (Table 4). Additionally, there was no statistical difference between the two groups in overall rate of unplanned re-exploration for bleeding (<24 hrs).

**Table 2:** Intraoperative variables

**Characteristic**	**Arista® AH (n = 103)**	**Control (n = 137)**	**p-value**
**Protamine to skin closure (min)**	93.4 ± 41	107.6 ± 56	0.02
**CPB time (min)**	114.3 ± 73	111.3 ± 77	0.76
**Arista applied (5 g applications)**	2.5 ± 1.7 (range 1–11)	N/A	--
**Intraop EBL (mL)**	1719 ± 1209	2024 ± 1503	0.08
**Intraop PRBCs (units)**	2.9 ± 2.9	2.9 ± 3.2	0.9
**Intraop FFP (units)**	2.5 ± 3	2.9 ± 2.8	0.28
**Intraop platelets (units)**	2.2 ± 2.5	2.2 ± 2.9	0.83

*PRBC* = Packed red blood cells; *CPB* = cardiopulmonary bypass; *EBL* = estimated blood loss; *FFP* = fresh frozen plasma.

**Table 3:** Postoperative outcomes

**Characteristic**	**Arista® AH (n = 103)**	**Control (n = 137)**	**p-value**
**CT output 48 hrs postop (mL)**	1594 ± 949	2112 ± 1437	<0.001
**PRBCs 48 hrs postop (units)**	2.4 ± 2.5	4.0 ± 5.1	<0.001
**-Patients received 1–2 units**	35 (34%)	56 (41%)	--
**-Patients received ≥3 units**	34 (33%)	49 (36%)	--
**ICU platelets 48 hrs postop (units)**	1.2 ± 2.3	1.8 ± 3.4	0.10
**ICU FFP 48 hrs postop (units)**	1.2 ± 2.6	1.9 ± 3.7	0.12
**ICU stay (median days)**	8(range 2–37)	9(range 2–57)	0.08
**30 day mortality, (%)**	17.5%	16.1%	NS

***CT*** **= chest tube;*PRBC*** **= Packed red blood cells;*CPB*** **= cardiopulmonary bypass;*EBL*** **= estimated blood loss;*FFP*** **= fresh frozen plasma;*ICU*** **= intensive care unit.**

**Table 4:** Postoperative complications

**Characteristic**	**Arista® AH (n = 103)**	**Control (n = 137)**	**p-value**
**Ventilator dependence**	21(20.4%)	33(24.1%)	NS
**Shock**	16(15.5%)	21(15.3%)	NS
**Renal failure**	17(16.5%)	23(16.8%)	NS
**Sepsis**	2(1.9%)	2(1.5%)	NS
**Stroke**	5(4.9%)	5(3.6%)	NS
**Unplanned re-exploration (<24 hrs)**	6(5.8%)	9(6.6%)	NS

**Figure 3:**
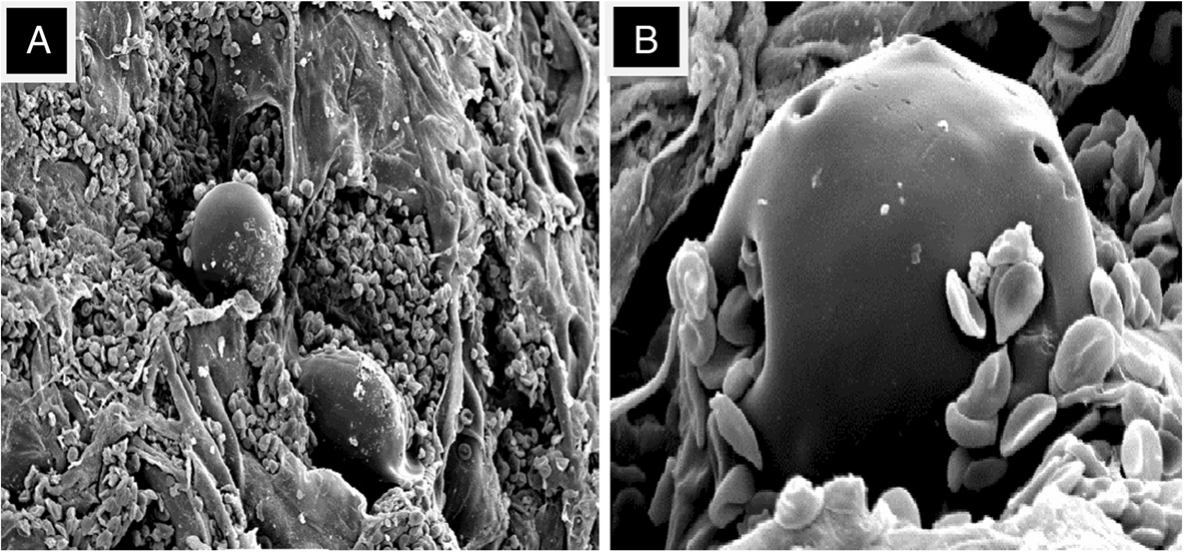
**Scanning Electron Microscopy (SEM) of Arista® AH post-application**. Note the presence of red blood cells and fibrin strands adhering to the Arista® AH polysaccharide hemosphere. **A)** Low magnification (450×). **B)** High magnification (1900×).

Intraoperative specimens of Arista® AH treated areas were obtained from the mediastinum of two patients who underwent LVAD implantation. These mediastinal clot specimens were obtained 10 minutes after the administration of Arista® AH as a broad field hemostat to the surgical field (Figure 3). Inspection of these high resolution images demonstrate Arista® AH microspheres interacting and appear to be “concentrating” the surrounding blood and clotting components. Additional investigative microscopy will be needed in future studies of Arista® AH to provide further clinical validation of the topical hemostatic mechanism of action.

## Discussion

The primary objective of this study was to evaluate the use of Arista® AH as a topical hemostatic agent in complex cardiothoracic surgical procedures requiring cardiopulmonary bypass in our surgical practice. Arista® AH was applied as a broad field hemostat to the surgical field to control active bleeding. To our knowledge, although a retrospective study, our experience with the use of Arista® AH is the largest case series reported for patients undergoing cardiothoracic procedures to date.

Our results demonstrated improved hemostasis with respect to hemostasis time, chest tube output, and need for PRBC transfusion in the first 48 hours postoperatively. However, we do acknowledge that chest output in the first 8–12 hours may be more meaningful as it relates to post-operative bleeding and there may be a serous component after 24–48 hours. Due to the retrospective nature of our study and availability of data, chest tube output was recorded at least on a 24 hour basis and earlier drainage was not available in all patients. Postoperative bleeding complications in cardiac surgery can have multiple etiologies. Invasiveness of the procedure, induced hypothermia, extended use of CPB, and increasing age, are major reasons for increased blood loss and the higher incidence of blood transfusions [6]. Concomitant postoperative complications, such as stroke, renal dysfunction, and systemic inflammatory response syndrome (SIRS) also increase incidence of postoperative bleeding [7]. Topical hemostatic agents are valuable adjuncts to cardiothoracic surgical procedures because they can decrease intraoperative bleeding, and may reduce the need for postoperative blood transfusion. In their study of 1,118 patients, Christensen et al. demonstrated that average hospital costs were substantially related to excessive postoperative hemorrhage in cardiac surgery [8]. Also in their study, postoperative bleeding was a significant risk factor for morbidity and mortality. Clinical interventions that can effectively prevent or address excessive postoperative hemorrhage in cardiac surgery are likely to have substantial cost-effectiveness potential. Their study also demonstrated that 22% of patients with excessive postoperative hemorrhage died compared with 6% of the patients without excessive postoperative hemorrhage (*p* < .0001). The potential to reduce postoperative bleeding, improve outcomes, and effectively reduce operative time/overall healthcare costs are major reasons that topical hemostatic agents are increasingly being used across all surgical disciplines. Although our study did not seek cost analysis, our data suggest that improved operative time and postoperative bleeding outcomes may translate into reduced healthcare costs. Additionally, although there was a decrease in transfusion requirements in the immediate postoperative period in the treated group, this did not translate into lower morbidity or mortality in this group, however larger numbers of treated patients in a future prospective trial may show significance in this very important area.

In a comprehensive review summarizing the efficacy of current topical agents, Barnard and colleagues concluded that bleeding during re-do sternotomy and the need for multiple blood transfusions resulted in poorer outcomes [6]. According to Barnard, “The ideal topical hemostatic agent would be one that is deployable even against brisk hemorrhage, which is independent of native clotting mechanisms and would not pass through salvage filtration systems while not being sourced from bovine or human origin”. In the current study, we demonstrated the safety and effectiveness of Arista® AH in cardiothoracic surgical procedures, and did not observe any adverse effects or reactions associated with its use. In a small case series, Tschan et al. demonstrated the safety of Arista® AH when employed in neurosurgery [9]. In their study, Arista® AH was used as an adjuvant hemostatic agent in 33 brain tumor resections. Hemostasis was achieved in 57 seconds on average. Moreover, at 3 months there was no product related neurological or embolic complications.

For ventricular assist device procedures, we leave the chest open for 24 hours after surgery and return the following day for washout and closure. For each of these cases, no Arista® AH was identified by visual inspection during 24 hours following the initial procedure. According to Hamdi, MPH is enzymatically degraded into water soluble fragments, leaving no trace of foreign material in as little as 12 hours, with a stable intact clot remaining [10]. In his work with thromboelastography, Dong states that the clot formed with expanded MPH beads, platelets, and clotting proteins has been shown to be more robust than a purely natural clot [11].

Prior to experience with Arista® AH in 2011, it was our surgical practice to use a topical hemostatic agent which included Gelfoam® and thrombin, Surgicel®, or FloSeal®. When we started using Arista® AH in our complex cardiothoracic procedures requiring cardiopulmonary bypass, we observed noticeable decreases in chest tube output and blood product transfusions in most of our patients. In addition, we noticed that we were spending less time in the operating room for hemostasis. From these earlier experiences with Arista® AH, we essentially changed our approach to surgical field hemostasis and started using only Arista® AH as our topical hemostatic agent (2011-present). In our current surgical practice for nearly every case, we also selectively use a sealant (CoSeal) on anastomotic suture lines, and after protamine administration, apply Arista® AH as a broad field hemostatic agent. Studies have revealed the use of effective surgical sealants for hemostasis at anastomoses and suture lines, and our study is not the first to investigate the potential of hemostatic adjuncts [12]. For example, Floseal® has been shown to reduce transfusions and post-operative bleeding in cardiac surgery [13]. However, there may be concerns with the use of Floseal® including cost, the biologic component exposure (thrombin), and its compatibility with cell salvage systems. We feel that the hemostatic agents we were using prior to Floseal® were more “localized” field hemostatic agents and the ability to broadly cover the surgical field was limited. We consider Arista® AH a “broad field” hemostatic agent, as it can be used to cover large areas within the surgical field. By broadly covering the surgical field with Arista® AH (a white powder), we were also able to identify additional localized areas of bleeding that potentially require additional suture placement. Therefore, with its ease of use and application, it can be readily available and deployed by the surgeon and operating room staff, especially when time is of the essence, and patients are bleeding.

In addition to complex cardiothoracic cases, Arista® AH has been used in the fields of obstetrics/gynecology, urology, otolaryngology, and general sugery, and has been shown to reduce intraoperative bleeding [14]-[17]. The big advantages of Arista® AH is that it appears to be safe and effective across multiple surgical disciplines, is completely absorbed in 24–48 hours, and contains no human or animal components. Furthermore, it does not appear to represent a nidus for infection or granuloma formation, and no adverse events have been reported in clinical studies or even in our own clinical experience [9],[14],[16].

### Limitations

There are important limitations that should be acknowledged, particularly the retrospective nature of this study, a highly select group of patients, and relatively short median follow-up duration. Also, all procedures were performed at a single center by two experienced surgeons and included the entire scope of our practice (cases requiring cardiopulmonary bypass) during the study period. The approach to this retrospective study was to include all cases requiring bypass (LVADs, transplants, cardiac cases), although subgroup analysis (i.e. assist device case, transplants, circulatory arrest cases, etc.) in a future trial with larger numbers of patients will be necessary. Multicenter prospective studies with longer follow-up will further establish the clinical efficacy of Arista® AH as a topical hemostatic agent in cardiovascular surgical procedures.

## Conclusion

The application of Arista® AH during cardiothoracic procedures resulted in a reduction in intraoperative hemostasis time, decrease in postoperative blood loss, and reduced need for blood product transfusion in the first 48 hours following surgery. Additionally, there were no adverse events associated with broad field coverage with Arista® AH within the cardiothoracic cavity. Although a retrospective study, our findings suggest that Arista® AH is safe and effective in reducing blood loss as an adjunct topical hemostatic agent in complex cardiothoracic surgical procedures.

## Abbreviations

MPH: Microporous polysaccharide hemosphere

SEM: Scanning electron microscopy

LVAD: Left ventricular assist device

SIRS: Systemic inflammatory response syndrome

CABG: Coronary artery bypass graft

CPB: Cardiopulmonary bypass

EBL: Estimated blood loss

PRBCs: Packed red blood cells

FFP: Fresh frozen plasma

CT: Chest tube

ICU: Intensive care unit

## Competing interests

This retrospective study was sponsored by C. R. Bard, Inc. (Davol), Warwick, RI. B.A. Bruckner, MD is a paid consultant for C. R. Bard, Inc. (Davol).

## Authors’ contributions

BB: Participated in the study design and drafted the manuscript and performed the operations. LB: Participated in study design and statistical analysis. LR: Participated in data collection. ES: Participated in data collection. UN: Participated in data collection and electron microscopy of patient samples. MR: Participated in review of manuscript. ML: Helped conceive study design and also helped perform the operations. All authors read and approved the final manuscript.
